# Spin-reorientation magnetic transitions in Mn-doped SmFeO_3_

**DOI:** 10.1107/S205225251700793X

**Published:** 2017-07-04

**Authors:** Jian Kang, Yali Yang, Xiaolong Qian, Kai Xu, Xiaopeng Cui, Yifei Fang, Venkatesh Chandragiri, Baojuan Kang, Bin Chen, Alessandro Stroppa, Shixun Cao, Jincang Zhang, Wei Ren

**Affiliations:** a Department of Physics, International Center of Quantum and Molecular Structures and Materials Genome Institute, Shanghai University, Shanghai 200444, People’s Republic of China; b Hangzhou Key Laboratory of Quantum Matter, Department of Physics, Hangzhou Normal University, Hangzhou 310036, People’s Republic of China; c CNR-SPIN, L’Aquila, Italy; d Shanghai Key Laboratory of High-Temperature Superconductors, Shanghai University, Shanghai 200444, People’s Republic of China

**Keywords:** spin reorientation transitions, rare earth perovskites, magnetic phase transitions, Mn doping

## Abstract

Spin reorientation is a magnetic phase transition in which rotation of the magnetization vector with respect to the crystallographic axes occurs upon a change in the temperature or magnetic field. For example, SmFeO_3_ shows a magnetization rotation from the *c* axis above 480 K to the *a* axis below 450 K, known as the Γ_4_ → Γ_2_ transition. This work reports the successful synthesis of the new single-crystal perovskite SmFe_0.75_Mn_0.25_O_3_ and finds interesting spin reorientations above and below room temperature. In addition to the spin reorientation of the Γ_4_ → Γ_2_ magnetic phase transition observed at around *T*_SR2_ = 382 K, a new spin reorientation, Γ_2_ → Γ_1_, was seen at around *T*_SR1_ = 212 K due to Mn doping, which could not be observed in the parent rare earth perovskite compound. This unexpected spin configuration has complete antiferromagnetic order without any canting-induced weak ferromagnetic moment, resulting in zero magnetization in the low-temperature regime. *M*–*T* and *M*–*H* measurements have been made to study the temperature and magnetic-field dependence of the observed spin reorientation transitions.

## Abstract 2

Spin-reorientation magnetic transitions are studied in Mn-doped SmFeO_3_.

## 1. Introduction   

The emerging demand for next-generation spintronic devices calls for more functional materials with outstanding properties. In this context, the *R*FeO_3_ (*R* = rare earth ion) oxide family with the *Pbnm* structure are promising candidates, in which not only the magnetic interactions between the 3*d* spins of the transition metals (Fe–Fe) but also those with the 4*f* moments of the rare earth ions (*R*–Fe) have important roles in their magnetic behaviour and magnetoelectric coupling (Zvezdin & Mukhin, 2009 ▸). Up to now, *R*FeO_3_ compounds have been investigated for future applications such as inertia-driven spin switching (Kimel *et al.*, 2009 ▸), laser-induced spin reorientation (SR) (de Jong *et al.*, 2011 ▸), ultrafast manipulation of spins through thermally induced SR transition (de Jong *et al.*, 2012 ▸), temperature and magnetic field control of SR (Cao *et al.*, 2014 ▸; Hong *et al.*, 2011 ▸; Cao *et al.*, 2016 ▸; Zhao *et al.*, 2015 ▸; Wu *et al.*, 2014 ▸; Wang *et al.*, 2016 ▸; Yuan *et al.*, 2013 ▸) and large rotating-field entropy change (Cao *et al.*, 2016 ▸; Huang *et al.*, 2013 ▸). The *R*MnO_3_ family also draws a lot of attention due to spontaneous electric polarizations induced by noncollinear spiral magnetic order (Kenzelmann *et al.*, 2005 ▸), a collinear *E*-type antiferromagnetic structure (Okuyama *et al.*, 2011 ▸) and strong antiferromagnetic pinning effects (Jin *et al.*, 2015 ▸). In *R*FeO_3_ and *R*MnO_3_ compounds, below their Néel temperatures (*T*_N_), the Fe^3+^ or Mn^3+^ sublattice will be ordered in a slightly canted antiferromagnetic configuration due to an antisymmetric exchange Dzyaloshinsky–Moriya interaction, while also showing weak ferromagnetism (Dzyaloshinsky, 1958 ▸; Moriya, 1960 ▸). Magnetoelectric (ME) coupling, which breaks spatial inversion and time-reversal symmetries (Mostovoy, 2006 ▸), has been the focus of extensive research to explore the mutual control of electric and magnetic degrees of freedom (Cao *et al.*, 2016 ▸; Fina *et al.*, 2010 ▸). Various magnetic phase transitions also occur in the presence of ME processes (Hemberger *et al.*, 2007 ▸; Kimura *et al.*, 2005 ▸) and dielectric properties may also change around the magnetic phase transition, thus providing an approach to trigger magnetodielectric coupling. It is worth noting that DyFeO_3_ has been found experimentally to have a field-induced gigantic ME effect (Nakajima *et al.*, 2015 ▸). Consequently, the modification of magnetic and electric polarization by controlling the phase transition is the main theoretical and experimental objective to realise new multiferroic materials that have application at room temperature.

One of the special magnetic phase transitions in *R*FeO_3_ which might be closely related to magnetically driven ferro­electricity is spin reorientation (SR). According to symmetry considerations and the antiferromagnetic nature of the coupling between the ions, there are three magnetic configurations allowed for the Fe^3+^ sublattice, namely Γ_1_, Γ_2_ and Γ_4_, with different major antiferromagnetic directions (White, 1969 ▸). Complex *R* ^3+^–*R* ^3+^, *R* ^3+^–Fe^3+^ and Fe^3+^–Fe^3+^ interactions can lead to competitive anisotropic magnetic features in *R*FeO_3_, possessing slightly different free energies when the magnetic moments align along the three main axes over varying temperature ranges. For *R*FeO_3_, the Γ_4_ spin configuration appears in the higher temperature region, while the other two spin configurations can be found when the temperature is reduced (White, 1969 ▸). At extremely low temperatures a different spin configuration may appear, due to the more complicated ordering of sets of rare earth ions, such as observed in TbFeO_3_ (Cao *et al.*, 2016 ▸) and DyFeO_3_ (White, 1969 ▸).

SmFeO_3_ has attracted much attention due to its intriguing behaviours, like fast magnetic switching (Jeong *et al.*, 2012 ▸), temperature-induced spin switching (Cao *et al.*, 2014 ▸), the highest SR temperature (*T*_SR_) among the *R*FeO_3_ system and its curious ferroelectric properties (Lee *et al.*, 2011 ▸; Kuo *et al.*, 2014 ▸). SmMnO_3_ is one of the *A*-type antiferromagnetic (AFM) *R*MnO_3_ compounds, showing negative magnetization (Cheng *et al.*, 2011 ▸) and magnetocapacitive effects (Jung *et al.*, 2010 ▸) as reported recently. In addition, the *R*Fe_*x*_Mn_1−*x*_O_3_ (*R* = Dy, Tb and Y) family has been studied a lot due to their varying physical properties and interesting phase transitions (Nair *et al.*, 2016 ▸; Mandal *et al.*, 2013 ▸, 2011 ▸; Hong *et al.*, 2011 ▸). Typically, the usual way to control the temperature and configuration of the SR transition is to dope other lanthanides into *R*FeO_3_, such as Sm_1−*x*_Dy_*x*_FeO_3_ (Zhao *et al.*, 2015 ▸) and Dy_0.5_Pr_0.5_FeO_3_ (Wu *et al.*, 2014 ▸). Here, we report a new way to change the SR transition by modifying the 3*d*–4*f* interaction between *R* ^3+^–Fe^3+^ with Mn^3+^ doping in single-crystal SmFeO_3_. By doing so, we found that the SmFe_*x*_Mn_1−*x*_O_3_ family can possess novel properties that are absent from the parent SmFeO_3_ and SmMnO_3_ rare earth perovskites. Here, we focus our investigation on the twofold SR magnetic transitions of SmFe_0.75_Mn_0.25_O_3_ and the continuously tunable phase transition as observed from the temperature dependence of the magnetization (*M*–*T*) under external applied fields and hysteresis loops (*M*–*H*).

## 2. Experimental   

The SmFe_0.75_Mn_0.25_O_3_ (SFMO) single crystal was grown by the optical floating-zone method (Crystal System Inc., model FZ-T-10000H-VI-P-SH). The compounds of the feed and seed rods were prepared by a solid-state reaction in which the stoichiometric starting materials were Sm_2_O_3_ (99.99%), Fe_2_O_3_ (99.9%) and MnO_2_ (99.9%) in a properly mixed proportion of 4:3:2. The temperature of the molten zone was controlled by adjusting the power of four lamps. The molten zone moved upwards at a rate of 3 mm h^−1^.

The crystallinity and crystallographic orientations were determined by X-ray Laue photography. The microstructure of the crystal was checked by X-ray diffraction using a Rigaku 18 kW D/MAX-2550 diffractometer (Cu *K*α radiation) with a scanning step of 0.02° and 2θ from 10° to 120°. The dc magnetization measurements were performed using a Physics Property Measurement System (Quantum Design, PPMS-9). Zero-field-cooling (ZFC) and field-cooling (FC) processes were used to acquire the temperature dependence of the magnetization.

## 3. Results   

**Figure 1:**
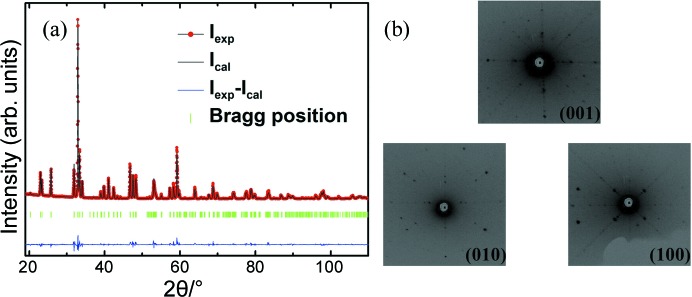
(*a*) X-ray diffraction pattern and (*b*) Laue back-scattering photography of SmFe_0.75_Mn_0.25_O_3_ single crystal along the (001), (010) and (100) crystallographic axes.

Fig. 1 ▸(*a*) shows the Rietveld refinement of powder X-ray diffraction (XRD) data from the single crystal of SmFe_0.75_Mn_0.25_O_3_ performed using the *AutoFP* (Cui *et al.*, 2015 ▸) and *FULLPROF* (Rodríguez-Carvajal, 2001 ▸) programs. The diffraction patterns can be assigned to the single-phase orthorhombic perovskite structure with space group *Pbnm* and no impurity phases were detected. The lattice parameters thus obtained are *a* = 5.39392 Å, *b* = 5.63707 Å and *c* = 7.67156 Å (*R*_wp_ = 0.133). Fig. 1 ▸(*b*) shows the clear Laue diffraction spots, indicating the high quality of our SmFe_0.75_Mn_0.25_O_3_ single crystal. Both XRD and Laue photography confirmed the excellent quality of the sample, and the cutting planes are precisely perpendicular to the *a*, *b* and *c* axes, respectively.

**Figure 2:**
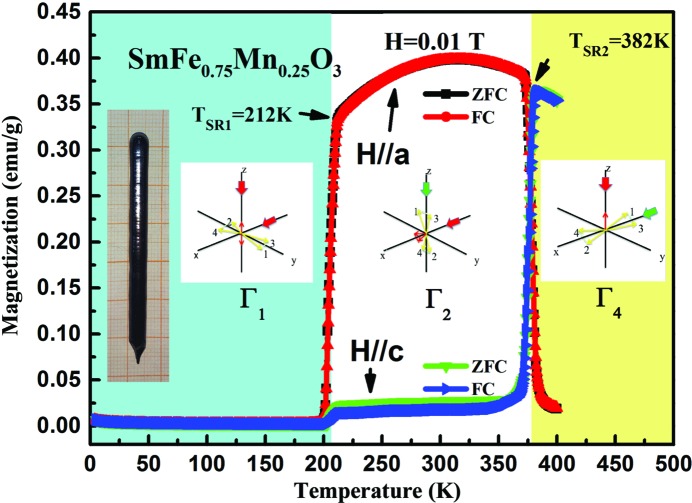
Temperature-dependent magnetizations of SmFe_0.75_Mn_0.25_O_3_ along the *a* and *c* axes under magnetic field *H* = 0.01 T. Three slightly canted antiferromagnetic phases Γ_1_, Γ_2_ and Γ_4_ can be distinguished by the shaded areas. The inset shows the SmFe_0.75_Mn_0.25_O_3_ single-crystal sample.

Fig. 2 ▸ shows the temperature dependence of the ZFC and FC magnetizations measured under a field of *H* = 0.01 T for the SmFe_0.75_Mn_0.25_O_3_ single crystal (sample shown in the inset) along the the *a* and *c* axes, respectively. The second-order transition starting from *T*_SR2_ = 382 K is the first spin reorientation, where the Fe^3+^ spins reorient from a configuration of canting antiferromagnetism along the *a* axis with weak ferromagnetism along the *c* axis, 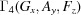, to canting antiferromagnetism along the *c* axis with weak ferromagnetism along the *a* axis, 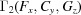. Another unique and new feature of the *M*–*T* curve is that at *T*_SR1_ = 212 K, the SmFe_0.75_Mn_0.25_O_3_ single crystal undergoes a second SR transition at a lower temperature with weak ferromagnetism along the *a* axis 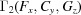, changing to complete antiferromagnetism with no net magnetization, 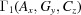. This kind of SR behaviour has never been reported in the literature to the best of our knowledge.

The transition over the whole temperature range might be called a Γ_4_ 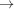 Γ_2_ 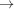 Γ_1_ double SR transition. Since pure SmFeO_3_ possesses a spin configuration from Γ_4_ to Γ_2_ due to the Sm^3+^–Fe^3+^ interaction at rather high temperatures (450–480 K), it is reasonable to have such a spin configuration transition in SmFe_0.75_Mn_0.25_O_3_ at a lower temperature of 361–382 K. The most interesting finding here is the spin configuration from Γ_2_ to Γ_1_ due to Mn^3+^ doping in SmFeO_3_ that gives a vanishing effective moment vector below *T*_SR1_ = 212 K. It is known that there is no SR in the whole family of the *R*MnO_3_ system. In addition, it is very rare for a rare earth orthoferrite to possess a Γ_1_ spin configuration, with the exception of DyFeO_3_ (Fu *et al.*, 2014 ▸), for which the SR transition temperature (*T*_SR_ = 37 K) is much lower than that of our single crystal here.

**Figure 3:**
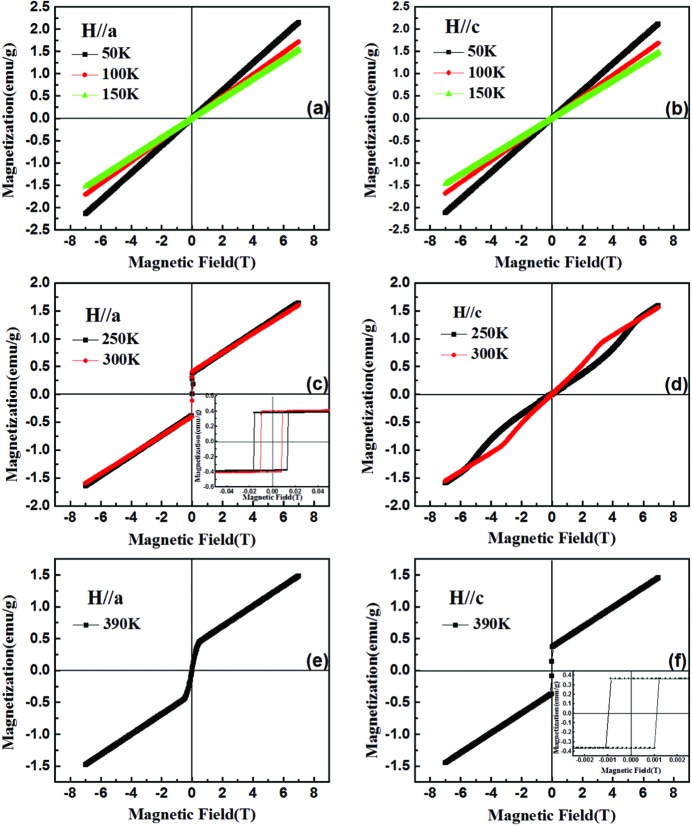
*M*–*H* curves for the SmFe_0.75_Mn_0.25_O_3_ single crystal along the *a* and *c* axes at temperatures of (*a*), (*b*) 50 K, 100 K and 150 K, (*c*), (*d*) 250 K and 300 K, and (*e*), (*f*) 390 K. The insets of (*c*) and (*f*) show enlargements of the plots at small magnetic field.

To investigate the SR transition further, the *M*–*H* curves were measured at different temperatures along the *a* and *c* axes, repectively. Figs. 3 ▸(*a*) and 3 ▸(*b*) show the standard linear AFM behaviour at 50, 100 and 150 K with a change of slope, which corresponds to the feature of Γ_1_ spin configuration below *T*_SR1_ = 212 K. Fig. 3 ▸(*c*) and inset show the uncompensated ferromagnetism component along the *a* axis, while Fig. 3 ▸(*d*) shows AFM-like behaviour but with noticeable kinks along the *c* axis. Above 382 K, the AFM-like and kink behaviour is more obviously seen in the *a* axis *M*–*H*, as shown in Fig. 3 ▸(*e*), while the *c* axis shows a ferromagnetism component in Fig. 3 ▸(*f*) and its inset. Inflexion behaviours are observed in Figs. 3 ▸(*d*) and 3 ▸(*e*) when the magnetic fields reach certain critical values, which suggests a field-induced SR. Therefore, according to Fig. 3 ▸, we can confirm that the weak ferromagnetism aligns along the *c* axis when the temperature is below *T*_N_ (around 600 K), and changes its direction to the *a* axis when the temperature drops below *T*_SR2_ = 382 K, before disappearing in any direction below *T*_SR1_ = 212 K, as shown in Fig. 2 ▸. Therefore, such a double SR transition induced by temperature is extraordinary for a single-phase rare earth perovskite.

**Figure 4:**
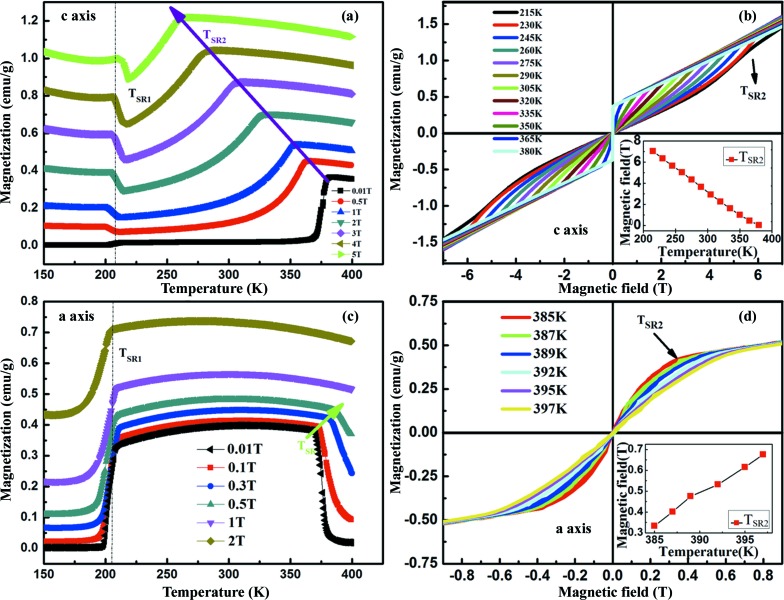
(*a*) *M*–*T* curves along the *c* axis at different magnetic fields from 0.01 to 5 T. (*b*) *M*–*H* curves along the *c* axis at different temperatures from 215 to 380 K. (*c*) *M*–*T* curves along the *a* axis at different magnetic fields from 0.01 to 2 T. (*d*) *M*–*H* curves along the *a* axis at different temperatures from 385 to 397 K. The insets in (*b*) and (*d*) show the linear temperature dependence of *T*_SR2_ along the *c* and *a* axes, respectively.

Fig. 4 ▸(*a*) shows the *M*–*T* curves along the *c* axis when we change the magnitude of the external magnetic field. Upon increasing the magnetic field, *T*_SR2_ moves to a lower temperature in a linear-like fashion. For example, we can see that the value of *T*_SR2_ decreases to 263 K under *H* = 5 T. Thus, we believe that when the magnetic field is high enough, the Γ_2_ spin configuration will be totally suppressed. On the other hand, *T*_SR1_ is found to be almost constant regardless of the magnitude of the external field. Fig. 4 ▸(*b*) shows the measured *M*–*H* curves at different temperatures ranging from 215 to 380 K. This diagram shows the evolution of the *M*–*H* curves with a change of slope around *T*_SR2_ at different temperatures, illustrating an intermediate state between two different magnetic configurations, corroborating the details of Fig. 4 ▸(*a*). Here, the magnetic order would be stabilized in a Γ_4_ spin configuration with a moment along the *c* axis after the intermediate state. Furthermore, we also observe from both Figs. 4 ▸(*a*) and 4 ▸(*b*) that, with increasing magnetic field, the inflection points move to low temperature. On the other hand, when we apply the external magnetic field along the *a* axis, Fig. 4 ▸(*c*) shows that the spin reorientation temperature *T*_SR2_ will move to the high-temperature region, as indicated by the green arrow. When the magnetic field exceeds 0.5 T, *T*_SR2_ moves above 400 K, which is beyond our temperature detection limit. Fig. 4 ▸(*d*) shows the *M*–*H* curves along the *a* axis at temperatures ranging from 385 to 397 K. The collected data show another linearly increasing behaviour for *T*_SR2_ *versus* magnetic field, consistent with that of the *M*–*T* curve in Fig. 4 ▸(*c*). In addition, *T*_SR1_ remains constant with varying magnetic field, as indicated by the vertical dashed line. To sum up, the Γ_4_ to Γ_2_ SR transition temperature can be easily controlled by magnetic field, whereas the Γ_2_ to Γ_1_ transition temperature is insensitive to the magnetic field.

## 4. Discussion   

As mentioned, the rare earth orthoferrites have two kinds of magnetic ion, *M* ^3+^ and *R* ^3+^. So there are three kinds of magnetic interaction, *M* ^3+^–*M* ^3+^, *M* ^3+^–*R* ^3+^ and *R* ^3+^–*R* ^3+^. These three interactions all consist of isotropic, antisymmetric and anisotropic-symmetric super-exchange interactions, which inevitably makes the magnetic properties of *R*FeO_3_ complex. Further, the antisymmetric and anisotropic-symmetric super-exchange interactions of *M* ^3+^–*R* ^3+^ are responsible for the temperature-induced SR (Yamaguchi, 1974 ▸). In our case, the interaction becomes even more complicated due to the doping Mn^3+^ ions and intriguingly introduces all three spin configurations allowed in *R*FeO_3_. By minimizing the free energy in the Γ_4_ to Γ_2_ SR transition with respect to θ and Φ (θ is the rotation angle of the easy axis in the *ac* plane and 2Φ represents the angle between two sublattices of the *R* ^3+^ spins), one can obtain the following equation (Yamaguchi, 1974 ▸):
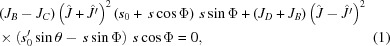


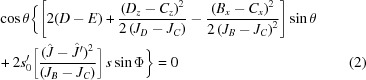
where *s* is the ratio of the mean values of the *R* ^3+^ and *M* ^3+^ spins, 〈*S*_*R*_〉/〈*S*_*M*_〉, which is the only parameter depending directly on temperature. The other parameters in equations (1) and (2) are different exchange constants. These two equations give the following three sets of solutions, (I), (II) and (III). 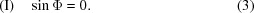
In this case, the stable angle of θ is 0 and this gives the Γ_4_ configuration in the high-temperature phase (382 K < *T* < *T*_N_). 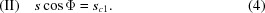
where 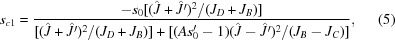
and 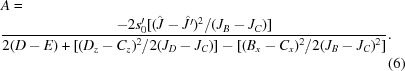
The equilibrium values of θ can be acquired as 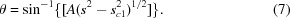
From equation (7), we can see that θ is zero when *s* = *s*_*c*1_ and will increase with increasing *s*. This implies rotation of the spin system. When *s* reaches another critical value of *s* = 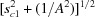, θ will take a value of π/2. This process corresponds to the continuous SR intermediate range (361 K ≤ *T* ≤ 382 K). 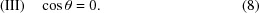
This indicates that the easy axis has rotated to the *c* axis and the system is in the Γ_2_ phase. Briefly, the case when *s* = 0 corresponds to the state at the Néel temperature. At high temperature *s* is small, and when *s* ≤ *s*_*c*1_ the free energy *F*(Γ_4_) is lower and the spin system will be stabilized in the Γ_4_ configuration due to the anisotropic energy of the Fe^3+^ single ions. As the temperature decreases, the sublattice moments of Sm^3+^ and Fe^3+^ will increase and *s* gradually approaches *s*_*c*1_ where the free energy *F*(Γ_24_) crosses *F*(Γ_4_). When *s* finally surpasses 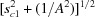, the free energy *F*(Γ_2_) becomes lowest, thus favouring the Γ_2_ configuration. This is the typical continous SR transition in most *R*FeO_3_ systems, but in the Mn^3+^-doped SmFeO_3_ single crystal the situation will become more complicated. Once the first SR transition is complete, the Mn^3+^ ions will be ordered with decreasing temperature and this will consequently influence the distribution of the free energy along the three main axes and *s* as well. When *s* reaches another critical value *s*_*c*2_, the free energy of the Γ_1_ configuration is smaller than that of Γ_2_ and Γ_4_, and the corresponding temperature is 212 K for our case.

So far, we have discussed two criticial values of *s*, namely *s*_*c*1_ and *s*_*c*2_. For an un-doped *R*FeO_3_ system, if *s*_*c*1_ ≤ *s*_*c*2_, a Γ_4_ to Γ_2_ SR transition will be seen, and if *s*_*c*1_ ≥ *s*_*c*2_, a Γ_4_ to Γ_1_ SR transition will occur (Yamaguchi, 1974 ▸). Indeed, if *s* never reaches *s*_*c*1_ or *s*_*c*2_ in the whole temperature range, no SR takes place, and this is the case for LaFeO_3_ and YFeO_3_. With the appropriate concentration of Mn^3+^, the influence of the Mn^3+^ sublattice will make *s* grow more rapidly as the temperature decreases and *s* will reach *s*_*c*2_ at high temperature where the spin structure transforms to the Γ_1_ configuration (Hornreich *et al.*, 1975 ▸).

The energy of an antiferromagnetic system in a magnetic field is lower when the antiferromagnetic vector is perpendicular to the direction of the field than when the two are parallel (Johnson *et al.*, 1980 ▸). A magnetic field applied along the antiferromagnetic axis of the Fe^3+^ system in the rare earth orthoferrites acts to cause reorientation of the Fe^3+^ antiferromagnetic vector to a perpendicular alignment. It is the competition between this Zeeman energy and the magnetic anisotropy energy of the Fe^3+^ system that determines the spin reorientation. In our case, since the antiferromagnetic vector of the Γ_1_ configuration is along the *b* axis, one would not expect a field-induced change in the SR temperature *T*_SR1_ when the external field is along the *c* or *a* axis. Accordingly, a field-modified spin reorientation is seen when the external field is along the *c* axis rather than the *a* axis, as the antiferromagnetic vector of the Γ_2_ configuration is along the *c* axis when the temperature is between *T*_SR1_ and *T*_SR2_. Weak magnetism along the *c*-axis direction can be induced by applying an external field along the *c* axis. The Γ_4_ configuration has its antiferromagnetic vector along the *a* axis, and an external field along the *a* axis can induce another Γ_2_ spin configuration when the temperature is between *T*_SR2_ and *T*_N_.

## 5. Conclusions   

In summary, we have successfully synthesized a single crystal of SmFe_0.75_Mn_0.25_O_3_, which has a single-phase perovskite structure, by the optical floating-zone method. The magnetic properties along different crystallographic axes have been studied in detail. Interesting double spin reorientation transitions Γ_4_ 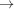 Γ_2_ 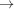 Γ_1_ were observed above and below room temperature. Field-induced spin reorientation has been investigated along the *c* and *a* axes in detail, showing the linear dependence and independence of SR temperatures *versus* magnetic field. Delicate interactions between the magnetic sublattices of Sm^3+^, Fe^3+^ and Mn^3+^ make this unique compound highly sensitive to magnetic field and to temperature. Due to its intriguing magnetic characteristics, novel magnetic switching devices could be designed based on this finding.
